# Exploring community resilience through Arctic residents’ narratives in the Republic of Sakha (Russia)

**DOI:** 10.1007/s13280-024-02071-y

**Published:** 2024-10-10

**Authors:** Natalia Doloisio

**Affiliations:** 1 Copernicus Institute of Sustainable Development, University of Utrecht, Utrecht, The Netherlands; 2 Center for the Study of the Sciences and the Humanities, University of Bergen, Bergen, Norway; 3 CEARC Research Center, Université Paris Saclay, Orsay, France

**Keywords:** Climate change, Community resilience, Narratives, Permafrost thaw, Republic of Sakha

## Abstract

This article uses the Resilient Community Development frame to explore how the residents from two coastal settlements in the Russian Arctic cope with the long-term effects of climate change, permafrost thaw and challenging socio-economic conditions. Semi-structured interviews were conducted in 2019 in Tiksi and Bykovsky (Republic of Sakha, Russia). These narratives were coded and qualitatively analyzed using the aforementioned framing as a guideline. This article shows that migration, mobility, culture, history, identity and extended networks play a key role in the way that these residents cope with disturbances and upheavals. Sharing past experiences and specific ways to interact with their natural environment, other neighbors and the land they live in are pivotal for the local social configuration and can underpin identity processes. This analysis demonstrates that residents’ narratives regarding socio-economic challenges, climate change and permafrost thaw can inform us about short-term concerns, but also how to enhance long-term community resilience.

**Supplementary Information:**

The online version contains supplementary material available at 10.1007/s13280-024-02071-y.

## Introduction

Climate change and its impacts are becoming increasingly visible. Such changes occur in constant intertwinement with major societal and environmental transformations (IPCC 2018; Nikulkina et al 2020; Da Cunha et al 2021). The Arctic region experiences more severe and rapid responses than the rest of the world (Ford and Furgal 2009; IPCC 2022) and is affected by the phenomenon known as “Arctic amplification”: the surface air temperature in this region has increased by more than double the global average over the last 50 years (Notz and Stroeve 2016). The annual mean Arctic temperatures between 2014 and 2019 have exceeded all records and keep increasing at more than four times the global average (Gong et al. 2023)). As a result, multiple complex systems are affected simultaneously, including the cryosphere, marine and terrestrial ecosystems and human systems (Doloisio 2022).

From a geographical perspective, the Russian Federation is the largest Arctic state (Rowe 2009). The Republic of Sakha is situated on the far northeast of the Russian Federation, and its entire territory is underlain by different forms of permafrost (Fedorov et al. 2018). It is known for its harsh continental climate and short temperate summers. It is the homeland to different indigenous peoples including the Sakha,^1^ Evens, Evenks, Yukaghirs, Dolgans and Chukchi. It is also the region with the highest share of indigenous peoples in the country (Fondahl et al. 2014). These indigenous populations are facing a rapidly fluctuating world. Conducting science with and for Arctic communities requires putting local residents—the specific forms of knowledge related to their lifestyle, their worldviews and ways of interacting with their surrounding environment, the rest of the community and their territories—at the core of qualitative research. Doing this can contribute to obtaining more legitimate, equitable and inclusive outcomes (Hauser et al., 2021; Yua et al 2022). Local residents identify and assess different processes derived from climate change and can establish complex causal relations between these with great detail (Galappaththi, 2019; Doloisio and Vanderlinden 2020; Hauser et al., 2021). In this context, narratives associated to changes have the potential to facilitate identifying local communities’ concerns, desires and priorities (Doloisio 2022) and to formulate policies, as well as adaptive and resilience-building strategies that are deemed legitimate and acceptable by local residents.

Narratives are stories that capture people’s experiences of living in a particular moment and context, as well as the relationships that emerge during the process (Clandinin and Caine 2013). They allow us to obtain a deeper understanding of the social configuration, the existing values (Da Cunha et al 2020) and to explore how questions related to meaning emerge (Blumer 1969) within each of the impacted communities. According to Wittmayer et al. (2019), narratives are language tools capable of structuring events and actions simultaneously. They are strongly culturally rooted (Herman 2003) and allow us to delve into perception, social representations, experiences, attributions and meaning regarding climate change (Doloisio 2022). This is why the social dimension of risks associated to climate change, including the way they are perceived and experienced by each community (Imperiale and Vanclay 2021), is essential, especially when evaluating the alternatives and types of action that local residents judge necessary and accessible to tackle rapid changes (Crate 2022; Doloisio 2022). Moreover, narratives can also provide useful information about the social configuration, deeply rooted structures and processes related to power relations, inequality and marginalization (Nohrstedt 2022).They can also contribute to unveiling local terminological specificities, establish causal relationships between processes/events and facilitate the interpretation of new emerging risk patterns—as experienced and discursively presented by local residents (Doloisio 2022). Addressing societal issues is essential when looking for new ways to conceive development and to address the effects of climate change using equality, sustainability and resilience as pillars (Thomalla et al 2018; Schipper et al 2020). Moreover, a narrative approach can contribute to avoid reinforcing colonial dynamics when conceiving ways-to-do-science, to produce and reproduce knowledge (Diniz De Figuereido and Martinez, 2021).

The Republic of Sakha (Russian Federation) has been inhabited from time immemorial and northern residents have developed multiple ways to adapt to such harsh climatic and environmental conditions. This article aims at obtaining a broader understanding of how residents from Tiksi and Bykovsky withstand the effects of rapid climate change, permafrost thaw and a challenging socio-economic context. More specifically, what elements are mobilized by the community to adapt and shape the future of their lives and livelihoods in alignment with their worldviews, knowledge and desires. Narratives from residents in Tiksi and Bykovsky addressing long-term processes such as mobility, culture, extended networks, ways of interacting with the surrounding environment can extend the understanding of the local social configuration and underpin identity processes. These play a key role in adapting to rapid changes and thus shape local forms of community resilience. In this sense, narratives are a twofold tool: on the one hand, they inform about the specificities of community resilience as experienced by residents from the coastal Russian Arctic, and on the other, these examples can also enhance community resilience applicability worldwide. Using Arctic narratives, it was possible to extend the scientific knowledge regarding the Resilient Community Development framing. This case study based in local narratives can contribute to reducing the overall vagueness often associated to the concept of “community resilience.” This article explores the dialogs between different fields and disciplines, and integrates rich qualitative information from the Bulunsky District into climate resilience research.

### Climate change and permafrost thaw in the Bulunsky District

Both climate and permafrost are central elements of life in Tiksi and Bykovsky and shape peoples’ identities, their ties with their territories and livelihoods (Doloisio 2022). People from northern settlements of the Republic of Sakha rely on frozen ground to build underground caves called ice-cellars where they stock fish and meat throughout the year. Traditional activities are also conditioned by permafrost: for instance, reindeers seasonally migrate to other lands and therefore require stable frozen soil. In winter, people as well as goods circulate between remote regions and settlements on ice roads (which run on a frozen water surface). Places of high spiritual or material value for residents are also built on permafrost, such as the coastal cemeteries of Bykovsky. Indigenous and non-indigenous residents from the Republic of Sakha described in great detail the wide range of manifestations of changes in climate and accelerated permafrost thaw and what this represents for their lives and livelihoods (Doloisio and Vanderlinden 2020; Doloisio 2022). For example, permafrost thaw increases the amount of work, time and money that people have to invest in ice-cellar maintenance. Additionally, reindeer herders can experience animal injury or death during the migration seasons. In face of accelerated coastal retreat, meaningful places may also be damaged or disappear, for example the “balok” (or Бaлoк in Russian)—a temporary house used as a shelter for hunters and fishermen during storms—or coastal cemeteries like the ones in Bykovsky.

Doloisio (2022) shows that climate change is often addressed by residents in the form of weather conditions including variations in temperature, snow, wind and seasonal length (which affects the duration of the navigation season). Warmer temperatures are reflected in two ways: (1) longer autumns which cause both a later freeze-up of water bodies and closing of the navigation season and (2) shorter winters which are linked with accelerated sea ice melting and an earlier opening of the navigation season. According to local respondents, winter roads—the main road for goods and supplies in the North—have been opening later causing delays in the delivery of fresh products to their settlements. Because of this, food security in the area could shrink while also increasing the pressure on accessibility to “black food” (meat and fish obtained by any mean). A later freeze-up of the rivers and lakes affects “Podledka” (winter-fishing from underneath the ice followed by immediately freezing the fish). Many fishermen mentioned suffering severe accidents because ice was not completely frozen. Nevertheless, the navigation season opening earlier could be beneficial to seaport activity, according to some locals. This would increase the amount of days where ships can navigate and distribute goods in Northern settlements. Local manifestations of changes in climate will continue shaping the current and future socio-economic development of the region and may also affect liveability prospects.

### Resilience from an Arctic perspective

Resilience is a concept that has been mobilized and defined by different disciplines and within different contexts. It has gained international popularity over the past decade, especially in fields such as urban studies, climate change adaptation and socio-ecological systems analysis (e.g., Turner et al 2022). Within social sciences disciplines, it has been used in issues related to children and families (Landau 2007; Peek 2008; Fothergill and Peek 2015), social problems (Clauss-Ehlers and Levi 2002; Doron 2005), class and urban studies (Sánchez-Jankowski 2008), rural sociology (Varghese et al 2006), among others. This concept is now being increasingly used in interdisciplinary work as well (IHDP 2005). While some definitions are oriented to promote scientific analysis, others try to tackle current and potential emerging problems (Arctic Council 2016). It is a concept that allows us to positively approach complex issues such as climate change, socio-economic issues, disaster management and other major multidimensional challenges. It is usually associated to enhancing sustainability and reducing vulnerability (Klein et al 2003). Moreover, because it is a broad concept with positive connotations, it can sometimes be used as a “bridging concept,” making it possible to gather different sectors, stakeholders and disciplines around a determined challenge (Wardekker 2018). This is why well-known international organizations have recently started to include this concept within their goals and agendas: for example, the UNFCC COP21 Paris Agreement (UNFCC 2021) and the United Nations Development Goals (UN, 2015), among others.

The flip-side of this concept is that there are many diverging definitions of resilience that focus on different aspects which may conflict and lead to a misinterpretation, therefore undermining its analytical applicability. People involved in projects or policies might have different ideas about what it entails or what the boundaries of the concept are. This can reflect theoretical and practical divergence (Kane and Vanderlinden 2015; Wardekker 2021). This concept has also been critiqued because it can sometimes be too theoretical (Klein et al 2003). Also, resilience of socio-ecological systems is generally linked to well-delimited territories (Forbes et al 2009; Forbes 2013; Crate et al 2017), which might narrow the potential to identify important relationships with external stakeholders and elements (Solovyeva and Kuklina 2020).

From an Arctic perspective, the concept “resilience” is often used as a guiding concept to delve into the multiple ways in which humans and their natural environment interact and how they cope with accelerated changes in the region (Vlasova et al 2020). It is possible to find models and framings that focus solely on one or just a few aspects of resilience. For example, the Arctic Resilience Interim Report (2013) proposed a system-based framing of resilience and defined it as systems’ capacity to cope with disturbances and recover in such a way that they maintain their core function and identity. It also relates to the capacity to learn from and adapt to changing conditions, and when necessary, transform (Arctic Council 2013). The Arctic is characterized by constant change (Arctic Council 2016, p. ix). Climate and permafrost are major drivers of change in the Russian Arctic and affect ecosystems but also shape northern residents’ lives and livelihoods (Doloisio 2022). Nevertheless, processes associated with migration, natural resources extraction, indigenous issues and socio-economic challenges, among others, are equally important in the North. This is why using one of these framings could lead to a limited or distorted understanding of key aspects of the social and cultural dimensions of resilience. In this sense, trying to understand community resilience from an Arctic perspective requires finding a bottom-up and holistic framing capable of addressing the underlying, complex and simultaneous interactions that exist between the physical, social and economic dimensions.

### Two remote communities within the Bulunsky district facing rapid changes: Tiksi & Bykovsky

**Fig. 1:**
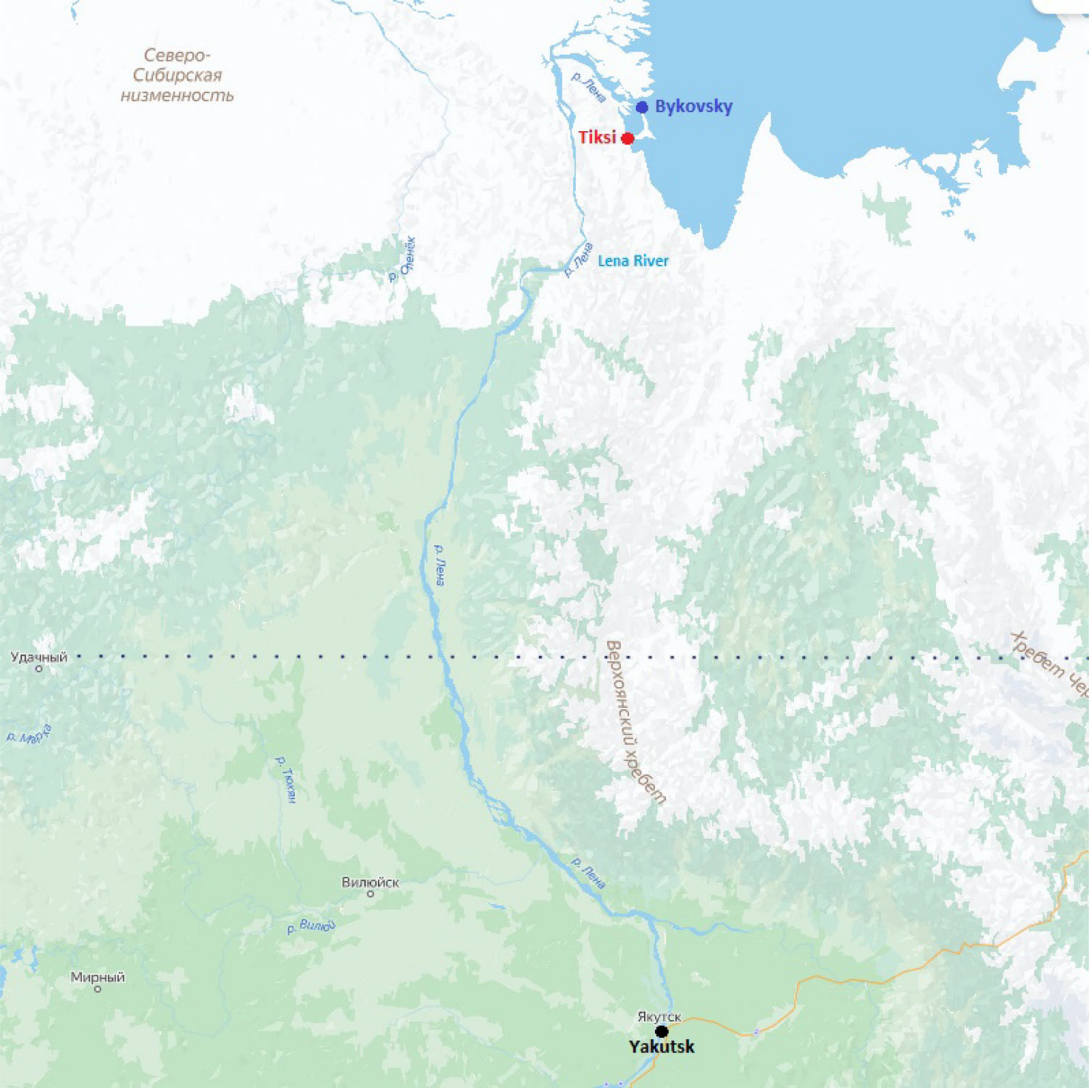
Map locating Tiksi and Bykovsky within the Republic of Sakha. *Source*: Yandex (n.d) retrieved May 1st, 2024, from https://yandex.ru/maps/?azimuth=0.0021621422254565204&from=tabbar&l=mrcpe&ll=125.277024%2C67.759379&z=5.2

Tiksi and Bykovsky are coastal settlements that are situated in the Republic of Sakha (Russian Federation) and more specifically, in the Bulunsky District (see Fig. 1 below). Bykovsky is 40 km north of Tiksi, located in Bykov’s Peninsula which naturally protects Tiksi’s harbor. Unlike Tiksi, Bykovsky has ice-rich sediments (known as Ice Complex) that are particularly affected by thermo-erosion and thermokarst processes (Meyer et al 2002). Tiksi is the administrative center of the Bulunsky District, while Bykovsky is an indigenous-fishing-oriented rural settlement. Tiksi was initially established in order to develop the Northern Sea Route. The sea port was and still is a key element of this settlement.

According to local residents, during the Soviet Union both settlements were prosperous and newcomers arrived from other parts of the country willing to develop them and enjoy the flourishing lifestyle that characterized the region. This came to an end with the Soviet breakup and the Russian Arctic underwent deep socio-economic modifications, leading, for example, to a radical shift in the directionality of the migratory fluxes (Doloisio 2022). For instance, Tiksi and Bykovsky experienced a sharp increase of out-migration (Table 1). Nowadays, they both share their remote emplacement within the vast Russian Arctic and are usually ruled by similar regional development and socio-economic policies/programs.

**Table 1:** Population in Tiksi and Bykovsky between 1989 and 2021

Year	Population Tiksi	Population Bykovsky
1989	11 649	627
2002	5873	363
2010	5063	517
2015	4557	523
2020	4793	514
2021	4173	513

Information obtained from: 1989 Soviet Census, 2002 and 2010 Russian Census, the 2019 Statistical Yearbook and the permanent population of the Russian Federation by municipalities as of January 1st, 2021

## Materials and methods

This article is the result of the analysis of qualitative information that was collected during the fieldwork conducted in 2019 in Tiksi and Bykovsky (Bulunsky District, Russian Federation). In 2018, I conducted fieldwork in Yakutsk, but the information collected in this opportunity was not included in this article (see Doloisio and Vanderlinden 2020). Intensive face-to-face and semi-structured interviews were conducted. The interview framework used as a guideline (Appendix S1) was written in English and then translated into Russian by our local colleagues. During the interviews, the French team represented by members from the CEARC research laboratory asked the questions in English, and these were simultaneously translated into Russian or Sakha by the local interpreters. Maintaining an open-ended method allowed me to integrate the constructivist grounded theory approach (Charmaz 2006). Using a flexible research design allowed exploring issues related to meaning, but also to obtain further information about social representations, experiences, perception and attributions (Doloisio 2022). Another fieldwork was planned for 2020: in collaboration with my Russian colleagues, I was to travel to Yakutsk to present the results of my analysis to local residents. Their feedback would be integrated as modifications to my research. This would have been a way to show respect and appreciation towards local residents who accepted to participate in this project. No one knows the reality they live in better than they do; consequently, new knowledge in this arena could not emerge without them. The fieldwork had to be postponed due to COVID-19 traveling restrictions and once again in 2022 when the war between Russia and Ukraine began. Within the SeMPER Arctic Project (Sense Making, Place attachment and Extended networks as sources of Resilience in the Arctic), it was intended to collect local stories related to changes, shocks, upheavals and their outcomes in three Arctic communities from Greenland and Russia. Due to the conflict between Russia and Ukraine, the planned fieldwork was officially cancelled. The research team had access to data initially collected in order to delve into the social and cultural impacts of climate change and permafrost thaw on the communities of Tiksi and Bykovsky. This corpus was rich enough to capture site-specific information that could also inform us about local forms of resilience in the Russian Arctic, as experienced and described by local residents. This analysis included eight interviews from Bykovsky and twenty-seven from Tiksi. Most respondents, from a variety of age groups, lived from traditional activities such as fishing, hunting, animal breeding or herding, which are the main economic activities of the Bulunsky District. In both settlements, several pensioners also participated in the interviews. In Tiksi, most interviewees held an administrative position and included lawyers, economists, heads of different departments, accountants, etc. There were also several interviewees who worked at the local library. In Bykovsky, respondents included local fishermen, workers from the kindergarten (teachers and the director), a specialist in housing and utilities services, etc.

Residents’ narratives were systematically analyzed in order to identify local forms or interpretations of resilience. In order to do so, I used the framework of resilience interpretations elaborated by Wardekker (2021) as a guideline. The Wardekker framework—which is based on an extensive review of resilience literature—is structured into two contrasted framings that are then integrated into a framing matrix: (a) short-term vs long-term and (b) system-based vs community-focused. In the following paragraphs I will refer to this categorization as Wardekker’s framework. Although other options could have worked, Wardekker’s framework seemed the most suitable for my research for several reasons: this article is a review that cross-examines resilience frameworks found in multiple resilience literatures that are relevant to urban climate resilience. He focuses on urban contexts, while covering multiple disciplines. The emerging tensions between short-term and long-term aspects, and community and systemic aspects described in the framework seemed highly relevant for Tiksi and Bykovsky: residents face many short-term challenges (e.g., damage of ice-cellars due to permafrost thaw) in the context of long-term changes (climate, socio-economic and demographic) that create new threats to their communities. Their resilience is determined by strong community dynamics (e.g., strong kin bonds) and the systemic context (e.g., influence of regional/national policies). So while this paper will focus primarily on long-term community resilience, the Wardekker (2021) framework that I present above helped me to remain aware of some other relevant aspects associated to resilience that I would have not been able to address with a narrower disciplinary framework. He also highlighted that research associated to this framing should address what and how communities desire to transform their environment for the future, but also the specificities related to how communities interact with their territories, with history, migration, culture, identity and extended networks. All of the aforementioned elements are related to the local characteristics of Tiksi and Bykovsky and to what local residents’ life experiences revealed as meaningful to them.

As a result of an extensive resilience literature review that considered different socio-ecological systems analysis, disaster studies, spatial planning, among others, this paper proposes four categories of resilience: Urban Shock-Proofing, Resilience Planning, Community Disaster Resilience and Resilient Community Development. By coding the narratives of local residents from Tiksi and Bykovsky, and subsequently creating code groups representing each of these four framings proposed by Wardekker (2021), it has been possible to link the codes and quotations with different elements and principles of resilience. Such principles were obtained from Wardekker (2021, Table 4). This table aims to demonstrate that the use of different resilience framings will have consequences for research, science-policy-society interactions, practice and governance. A set of resilience principles are therefore listed to exemplify the specificities of each framing. An initial analytical round mobilized the four different framings. A focus was put thereafter solely on the Resilient Community Development framing (long-term evolution, people & communities) as this was one of the framings that predominated within the corpus. According to Wardekker (2021) this framing is still underdeveloped and underrepresented within scientific literature. Analyzing what it means to be a resilient community from an Arctic perspective can also contribute to identifying important elements that have a central role in practical long-term processes and that might be undervalued or overlooked by research projects and policy interventions. Considering the specific ways in which the material (e.g., infrastructure, winter roads) and non-material dimensions (e.g., culture, identity processes) interact in the Russian Arctic, it is essential to pay attention to each of the participants, processes and elements. In this sense, using a framing such as the Resilient Community Development will make it possible to integrate them and enhance the understanding of how migration, traditional knowledge, extended networks, sharing a common past and solidarity can contribute to counterbalancing the effects of accelerated permafrost thaw and changes in climate.

### Coding

Wardekker (2021) focuses on the analysis of two different framings of resilience (Equilibrium/Evolutionary and Systems/Community) which propose different perspectives and practical implications for resilience-building. Equilibrium resilience focuses on short-term and acute stressors, while Evolutionary resilience is associated to long-term changes. System resilience focuses on maintaining the functions of the city as a system and the well-being of their citizens. Community resilience addresses issues related to community well-being and social cohesion, among others. He cross-examined and integrated these framings, obtaining a matrix with four new categories of application: “Urban Shock-Proofing” (short-term equilibrium, systems), “Resilience Planning” (long-term evolution, systems), “Community Disaster Resilience” (short-term equilibrium, people & communities) and “Resilient Community Development” (long-term evolution, people & communities). Each of them adopts different approaches, defines what is relevant differently, uses different notions and mechanisms and proposes different ways to involve participants (Wardekker 2021).

**Fig. 2:**
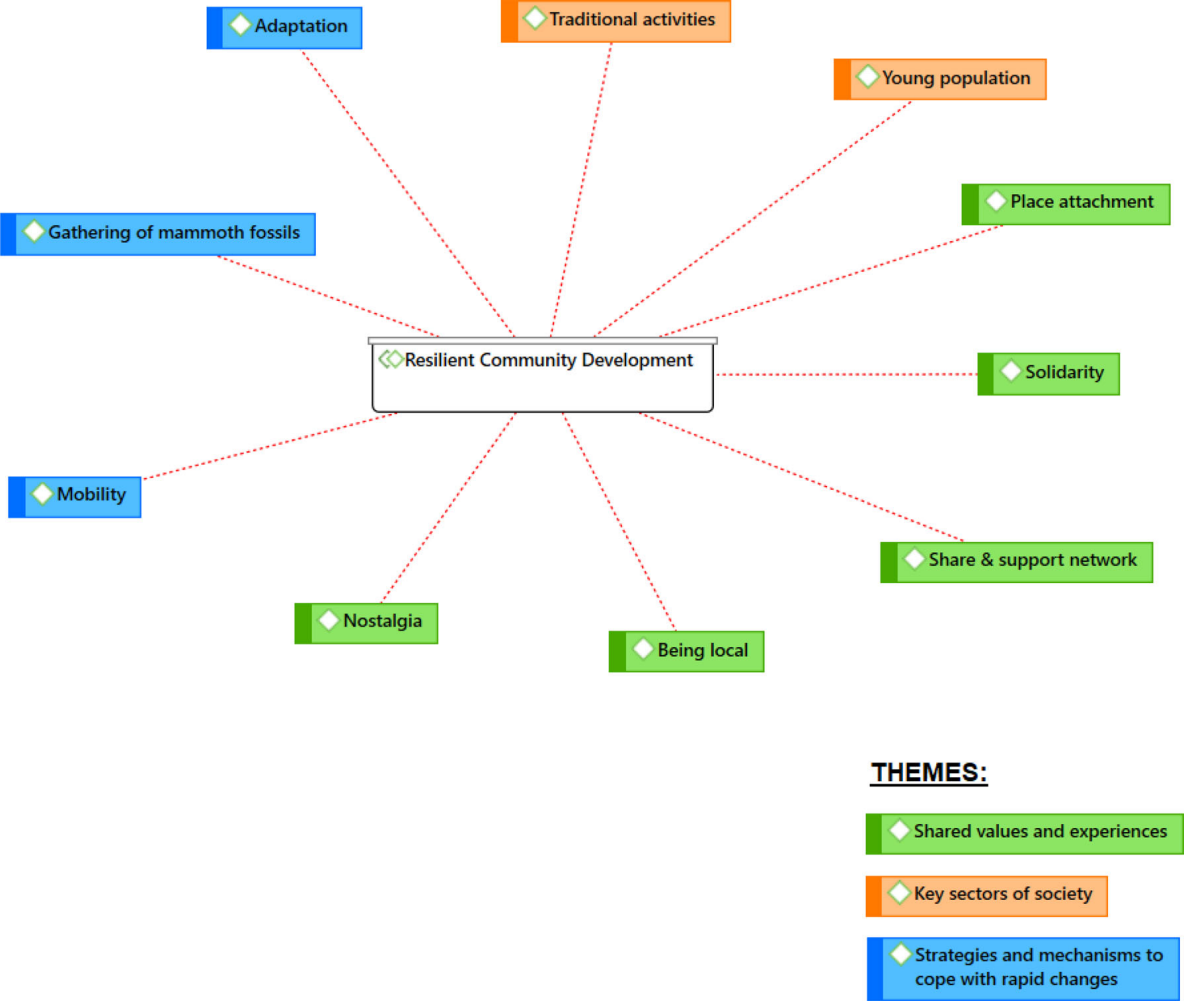
Schematic representation created with Atlas.ti. It shows the ten main analytical codes associated to the Resilient Community Development framing. It also shows which codes are associated to each of the three themes that emerged from the analysis

Although this framing analysis was oriented toward urban contexts, this study explored the theoretical bases of each of them to determine if these categories could also be helpful within an Arctic context or if new categories should be considered. For this purpose, the second stage of this research included creating four code groups in the hermeneutic unit. These were: “Shock-proofing,” “Resilience planning,” “Community disaster resilience” and “Resilient community development.” Each code with its associated quotes was linked to some of these code groups. Moreover, a schematic representation was produced where it is possible to visually identify the existing ties between each group code and their associated codes and quotes (Fig. 2). This article will focus on the Resilient Community Development code group. It will analyze how it has been associated to ten different codes from the hermeneutic unit, with its associated quotes and resilience principles. Using resilience principles can facilitate the interpretation of how narratives relate to local forms of community resilience. These principles describe specific mechanisms or aspects that underpin resilience. Therefore, they can be used to identify in a structured way, how and why an observation might impact resilience. The combination of narratives and principles can contribute to formulating solutions that are grounded in local reality and residents’ desires and actions.

## Results

From the qualitative analysis of local residents’ narratives, it was possible to produce a schematic representation (see Fig. 2 below) that links the Resilient Community Development framing with each of the ten analytical codes. This facilitates identifying the existing ties between this specific code group and the codes. At this stage, some thematic patterns based on points in common existing between the codes also emerged, allowing me to create three different themes: “shared values and experiences,” “key sectors of society” and “strategies and mechanisms to cope with rapid changes.” This is why the results will be presented on tables that regroup codes under these three themes and include their associated quotes, resilience principles and the corresponding analysis.

- The codes associated to the first theme emerged from feelings or meaningful experiences that were evoked by interviewees. Including feelings and emotions in this analysis is relevant since it reflects what respondents described as meaningful to them. Moreover, these are often overlooked in resilience frameworks.
- The second theme included two sectors that respondents identified as central in the development of life in northern settlements.
- The third theme gathers codes that, unlike the first one, are strictly related to action: adapting, gathering mammoth fossils or moving.

Some of the codes presented here organically required more attention because of the deep and extended implications for local residents, and therefore, for the overall analysis. The interpretation of these thematic patterns will be addressed under each table. I argue that this step of the research could enrich the understanding of the different pillars of community resilience using the life experiences and perceptions of residents from Tiksi and Bykovsky as an entry point.

Table 2 includes the codes “being local,” “solidarity,” “nostalgia,” “place attachment” and “share and support network” which inform us about shared feelings, values and experiences that reinforce local residents’ sense of belonging and often explains their reluctance to out-migrate. They provide rich information about the unique ways in which residents interact and develop bonds both with the territories and with other people, while stressing the central role that history has played in the common memories at a local and regional scale. They all contribute—to some extent—to counterbalance the challenges that the respondents described (for example, high prices of goods and services, low wages and unemployment; accelerated environmental changes, etc.). In this sense, it is possible to infer that they can enhance adaptation and therefore, also be key determinants in long-term community viability.

**Table 2:** It contains five codes that are related to shared feelings, values and experiences that help residents counterbalance the experienced challenges in Tiksi and Bykovsky

Shared feelings, values and experiences	
1. **Being local**^a^	
*Associated resilience principles:* learning and flexibility; social connectedness community robustness	
Analysis	Associated quotes
“Being local” is a concept that often came up during interviews conducted in Tiksi and Bykovsky, but not in the sense that our interviewees’ lives or experiences are circumscribed by restricted horizons or boundaries (Ingold 2015). “Locality” is in these cases based on elements such as a shared identity, a particular sense of mobility, common customs (e.g., low tolerance to hot weather) and kinship ties, specific knowledge related to nature which emerge in their daily lives, among others. This is why, “locals” not only include indigenous peoples, but also anyone born in Tiksi, Bykovsky or neighboring settlements who share a particular way of living, understanding and interacting with their surrounding environment Since locality is rooted in a particular form of belonging that is related to site-specific social and historical processes and environmental conditions, this could enhance coordinated and faster action between residents in face of accelerated changes. More specifically, having an extended common understanding of how to live “as a local” can also enhance unity when it comes to evaluating what actions are desired by local resident to tackle new emerging risk patterns (decision-making) and therefore, increase their self-determination	INTERVIEW 4 QUOTE 29–3: “Locals [are better adapted], it seems to me. My mother is local, and when it was around + 20/25 °C, she barely survived. Well, she barely could. She got used to the cold. She is accustomed to a cold summer in Tiksi. And when it was hot, everyone was like: "When will it be cold?" (…) Apparently, I'm local too, because I can’t stand heat, I need cold. I'm from Yakutsk, I'm not local (laughs) (…)” INTERVIEW 13 QUOTE 7: “I'm local, Evenki, Indigenous. I was born here. During childhood I was in the herds. I grew up there. I studied in the school of Kyusyur from which I graduated (…) I am a Commodity expert of industrial goods. Well, I grew up in the tundra since childhood. It’s very beautiful.”
2. **Solidarity**	
*Associated resilience principles:* community robustness; social connectedness	
Non-material aspects like the broadly extended solidarity and reciprocity relationships between Northern residents are essential to understand the social implications of climate change and how resilience is locally experienced. This particular kin-based connection does not rely on previous contributions but instead is a form of mutual aid based on a spirit of strong solidarity and reciprocity. Climatic conditions and remoteness have historically contributed to embrace this philosophy which aims to voluntarily care for and support each other with what each member has to offer. This can enhance local forms of social and cultural capital, which are involved in long-term processes and are often overlooked in the resilience literature	INTERVIEW 29 QUOTE 72–1: “(…) Our people they live in old good times. We help each other. We support newcomers and treat them. We can just give it to him. We will not sell it. We will just give it. It is a tradition.” INTERVIEW 2 QUOTE 244–5: “(…) Because the distances are so big here, we cannot live without mutual support. For example, the displaced people from Churapchinsky district^b^. They mostly died at the time when they were sailing [traveling down the Lena River]. And when they arrived here…everyone supported them and they all lived (…)”
3. **Share & support network**	
*Associated resilience principles:* partnership; wellness; collective action; strong regional identity	
The narratives from residents from Tiksi, Bykovsky and Yakutsk, demonstrated that in the Republic of Sakha exists a very unique kin-based network that contributes to counterbalance the effects of high prices of goods and services and relatively low wages. This happens at two levels: between residents from settlements/villages and between urban dwellers and residents from settlements/villages. The second example is what Argounova-Low (2007) describes as “close relatives and outsiders” relationship. Within this network, friends and relatives can exchange material and non-material things. For example, Northern residents from Tiksi or Bykovsky living from traditional activities such as hunting, reindeer herding and berry gathering, send meat, fish and other products to their relatives and friends from southern latitudes (e.g., Yakutsk). In exchange, their urban relatives provide them help in other forms such as completing administrative procedures or helping their children in educational matters such as entering University. These dynamics can also be found between residents from different Northern settlements, for example: people from Bykovsky and Tiksi obtain reindeer meat from Namy (situated on the river) and Naiba (on the coast). Friends and relatives from Tiksi help them with administrative procedures and residents from Bykovsky provide them with fish. This network based on mutual aid and support is intrinsically rooted on a spirit of strong solidarity and reciprocity (Doloisio 2022)	INTERVIEW 2 QUOTE 6–1: “Life in the village is calm and even. In fact, all people support each other. Because they are separated from everything (…) Human qualities are very much appreciated. Helping each other. Well, the life of residents is directly dependent on natural conditions.” INTERVIEW 8 QUOTE 117: “Well, we obtain meat, fish in advance every summer—friends and relatives give provide us. And so from the store you can buy pasta, fruit, butter.”
4. **Nostalgia & Soviet times**	
*Associated resilience principles:* wellness; Engagement; Partnership	
Locals’ narratives demonstrate that historical processes and identity issues are at the core of their conception of livability. The word “nostalgia” repeatedly emerged in locals’ narratives when talking about “old times” (here used in most cases as a synonym of “Soviet times”) to describe a specific way of feeling about what this region used to be like during Soviet times, but no longer is. Soviet nostalgia appears as a point of reference that is used by residents (especially elder people) to compare their current living conditions. More precisely, residents associate it with: - transportation to and from the North that was more accessible. For example, there were direct flights between Moscow and Tiksi - a broader diversity of products available - the presence of qualified professionals and high quality healthcare - obtaining higher wages, earlier pension entitlements and subsidized vacations This prosperity came to an end after 1992 when the liberalization of prices led to a sharp increase in the prices of goods and services in the North	INTERVIEW 13 QUOTE 12: “Well, the main thing with us (Tiksi) is the sea. Which has led and is leading so far. All sorts of cargo. Now there are less, there are less now. Earlier in Soviet times, there was generally beauty. We had everything. And now, look, everything is somehow not very … And there were a lot of ships, and now there is not a single ship. Everything was cheaper here.” INTERVIEW 34 QUOTE 9: “Earlier in Soviet times everything was good. Provisions came from Moscow and from St. Petersburg, Leningrad. First priority. The food was very good. Clothes, everything. The seaport worked well. Then, during perestroika, in the 90s, when everything came to ruin and the seaport was closed. People began to leave, experts left.”
Most respondents described feeling nostalgia while expressing their strong desire to see their settlements be “rebuilt like in the past times.” This past reference provides them with an “ideal” of how they would like to see their settlements look like in the future. Sharing that feeling gives to residents the feeling that they have something worth fighting—or potentially staying for -. The memories of what it once was, are a reason for many locals to feel “proud” of living there despite of the current challenges. Residents also mentioned observing “signs of development” in the region—related to new investments—which makes this ideal to be perceived as achievable and not as an imaginary anymore. The aforementioned elements fill residents with optimism and therefore “nostalgia”—as they conceive it—deserves to be interpreted as feeling that enhances resilience	
5. **Place attachment**	
*Associated resilience principles:* wellness; community engagement; community agency; social connectedness	
In opposition to the widely spread popular saying that Tiksi might become a ghost settlement, narratives collected in Tiksi and Bykovsky revealed different manifestations of place attachment among local residents. Human bonds and closeness, a deep sense of familiarity and an appreciation for the biophysical environmental seem to be some of the main elements that shape attachment to place in these settlements. Most of them emerged when respondents were analyzing livability in these settlements and future perspectives. In fact, the adverse socio-economic conditions and rapidly fluctuating environmental and climatic conditions force northern residents to consider what their options are: should they leave or should they stay? For some, the idea of moving out to other regions of the Republic of the country do not seem to have deeper emotional implications. However, others explicitly manifested their reluctance to leave. In such cases, respondents evoked different elements and mechanisms that help them counter-balance the negative effects of the current living conditions. Giuliani (2003) underlines the importance of bonds with places since it determines human groups’ existence. According to her, relatives, friends, places of worship, workplaces, leisure places provide specific values, common goals and meaning and reinforce identity processes	INTERVIEW 18 QUOTE 13: “People who grew up here in Yakutsk, it’s pretty easy for them to come and go. My classmates from Tiksi and from Siktakh need to go back home. Every summer, I come back. Its love for your birthplace maybe. It’s not similar for people who grew up in bigger cities. The negative point is that there isn’t a lot of people. So when moving to another place, it’s really hard, you need to introduce yourself, to know how to socialize. Talk with people again! Tiksi is really small and we know each other, it is simple to meet people, you come out and say hi! (…)” INTERVIEW 16 QUOTE 1: “Tiksi is my home, I love it so much. If I could, I would like to come back to Tiksi because I feel a responsibility for the whole land. That is why I want to work for my home, for the people who live there. (…) Infrastructure is really bad, internet (he laughs) even worse! By the way, I think that in the future it will be better because the Arctic is an area of interest for the government. It will take time.”

^a^ “мecтнoe нaceлeниe” in Russian

^b^They were displaced from Central Yakutia due to the drought and starvation. The government forcibly displaced them to Bykovsky to work for fishing state farms. More fishermen were needed in this settlement during the World War II and the consequent food crisis

**Table 3:** It includes codes that are related to key sectors of the society

Key sectors of society	
*Associated resilience principles:* wellness; social connectedness; knowledge building; resource engagement; community robustness	
6. **Traditional activities** (includes the following codes: “berry gathering,” “fishing” and “hunting”)	
Analysis	Associated quotes
Traditional activities are at the core of the Northern lifestyle and identity. They dignify residents’ lives by increasing their feeling of well-being and give them a unique sense of belonging. By fishing, hunting and gathering berries, locals are not only capable of providing traditional food for their friends and relatives—even those living in distant places like Yakutsk—but also enhance their sense of well-being. In Yakutian “black food” refers to hunting-food (fish and meat). Similarly, “white food”—refers to dairy products and “ground food”—food that is gathered (berries, mushrooms, herbs). Each of these products is associated to a specific form of traditional knowledge and ways of interacting between residents and the natural environment, which are also related to their Worldviews and the unique climatic conditions that they live in. Moreover, traditional activities and having access to natural resources also contribute to reducing the negative impacts of low wages and high prices in northern settlements like in the Bulunsky District. Practicing such activities also allow people to access traditional food at a lower price than if they were to buy them in a shop	INTERVIEW 29 QUOTE 95–1: “[…] of course! We have been fishing since childhood. My children were fishing in summer with a seine. In winter and in summer […] yes, we go fishing with our husbands, to help them […] they start fishing after primary school I think. When they turn 10 or 12.” INTERVIEW 29 QUOTE 69–9: “I am often asked if the fishermen deliver all fish that they caught. I say:” Of course, not. Where would they buy that fish? The fish that they caught? They deliver the part of fish according to the plan^a^. They keep part of fish for their families. It is not only for food. They can exchange it for something, for example. Their salaries are too small. Fish is not only the basis if the salary. It is the basis of well-being. It is for food and other things […] As long as you have the black food—you are alive”—so to say. You are replete.”
7. **Young people**	
*Associated resilience principles:* human resource development; collective action	
The presence of young people in the North is perceived by local residents as positive as this creates a more stable demographical composition and a broader network of professionals in these settlements. In this sense, a respondent from Tiksi mentioned that the absence of young people in Tiksi is a problem as according to them, this is directly related to the lack of specialists, for example, doctors (interview 15 quote 36–2). During the Soviet days, these was not a problem. However, another interviewee mentioned that the out-migration of young people seems to have stopped a few years ago (interview 19 quote 32–8)	INTERVIEW 15 QUOTE 36–2: “Yes (it is a problem that young students leave Tiksi), because we need young specialists, but there are none. Of course. Cultured, well-mannered, educated people. Yes, the level is, indeed, dropping.” INTERVIEW 19 QUOTE 32–8: “Yes, many (students who study abroad) do not return. There is a problem. Young people now have one criterion: somewhere there (let's take the same Yakutsk)—where the Internet is better. And due to such small pluses—children make their choice, let’s say so (giggles). Well, they’re coming back little by little anyway. The very outflow, mass migration, has stopped (…) I think that over the past 5 years, the situation has stabilized a little.”
In Bykovsky there are no major changes in terms of migratory processes of young people. The average age is 36–37 years old and most men are involved in fishing. The Artel Arktika (ex-Soviet Collective farm that then became a private cooperative association) continues its fishing activities and provides jobs to the majority of the men	
The Russian government has shown its geopolitical interest in its Arctic territories and its willingness to develop them. This could lead to an improvement in the infrastructure and socio-economic reality in the Russian North and therefore, increase the attractiveness of the region for young people. The “Strategy for socio-economic development of the Arctic Zone of the Republic of Sakha until 2035” which was signed in 2019; the “Comprehensive Plan of Development of Tiksi for the Period up to 2025” which was signed in 2020 (Kondrateva 2017) and the Strategy for the Development of the Russian Arctic Zone and National Security until 2035 (which was signed in October 2020) are some examples of the Russian government efforts to foster the socio-economic development in the region, which would also contribute to attract young people to return to their settlements of origin. Recently graduated professionals who come back to Tiksi can also foster the stabilization of the population and contribute to establish a solid network of professionals (which is a major concern for elderly people), among others	

^a^The local post-Soviet fishery enterprise formulates and gives to its employees a work plan which establishes how much fish they should catch per season

The codes “traditional activities” (which conceptually include fishermen, hunters, animal herders/breeders) and “young population” refer to two sectors of society that play a key role in the current social configuration and in shaping the future of these settlements (Table 3). The climatic and environmental conditions of the region, the remoteness of these settlements regarding big urban centers like Yakutsk or Moscow, and the cultural heritage place fishermen, hunters and animal herders/breeders as one of the most relevant actors at a local and regional scale. They are in charge of obtaining and distributing the base of traditional food: “black food” which in Sakha refers to meat and fish. By doing this, they enhance their own feeling of well-being and also perpetuate specific forms of knowledge and ways of existing in this world throughout generations. Improving living conditions in the North (for example, by extending the Internet network) could increase the younger generation’s interest in continuing living in a traditional way which is essential in order to stabilize northern population in the long-term, as well as to guarantee the survival of traditional knowledge and ways of living.

To finish with, Table 4 includes the codes “adaptation” “gathering of mammoth fossils” and “mobility” that refer to different strategies or mechanisms that local residents mobilize to tackle rapid and unprecedented changes in their settlements and district. These demonstrate that residents in the Bulunsky District have historically found—and keep finding—multiple and diverse ways to attenuate the undesirable effects of changes in climate, permafrost or the socio-economic reality they live in without any external input. The three of them are undoubtedly conditioned by the geomorphological, geographical and culturally specific characteristics of the Bulunsky District.

**Table 4:** It includes codes that are related to different strategies or mechanisms that residents use to counterbalance the challenges that they are currently experiencing

Strategies and mechanisms to cope with rapid changes	
8. **Adaptation**	
*Associated resilience principles:* Diversity; preparedness; knowledge building; collective action	
Analysis	Associated quotes
The word “adaptation” was far familiar to local residents. However, this was used in different contexts and with different implications, according to the type of experiences and knowledge of the respondent	INTERVIEW 15 QUOTE 24–5: “A human is such a creature, that adapts to everything (…) it seems to be that everyone can adapt” INTERVIEW 8 QUOTE 88: “Well, here the northern residents probably. They will not be cold. Heat too could probably be better. Well, reindeer herders in the first place. They live there in yurts, not in houses. Everything is manual. Everything is developed from improvised means (…).” INTERVIEW 12 QUOTE 17–5: “Well, I will do well, because I have a husband who grew up in the village, lived on the fishing-ground and fished. He knows everything: fishing, hunting, and so on. We will have no problems with this, I believe. And those who are accustomed to comfort, constant life in apartments with modern amenities and so on. It seems to me that (…) it will be more difficult for them. And for those who came here—who are not natives, nor locals. There are many newcomers who live and work here. I think it will be more difficult for them. “
A respondent pointed out that “locals”—people from the District—would be the most capable to cope with changes associated to climate and permafrost (see code “being local”). This naturally places them in an opposite group—people from southern latitudes -. The expression “being local” here exceeds the mere fact of being born in a determined latitude, but instead is used as a concept that extends to also having specific forms of knowledge and ways of interacting with the natural environment that according to these respondents, can enhance adaptation within the current rapidly changing context. When it comes to climate change, other respondents mentioned that “northern residents,” and more specifically reindeer herders will be more capable to counterbalance the negative impacts of a warmer weather (see interview 8 quote 88). Another woman mentioned that knowing how to practice traditional activities will increase the possibilities of her household to adapt to changing conditions (see interview 12 quote 17–5)	
Traditional activities and the associated knowledge are transmitted between generations and are deeply rooted in the specific human–environment interactions that have been developed in this region. Technical and material forms of adaptation were also mentioned but will not be included in this analysis as they seem to be related to a strong reliance on external funding to cover expenses	
The fact that residents from Bykovsky openly addressed and reflected on the possible implications of being forcibly displaced due to accelerated coastal erosion, demonstrates a flexible mindset and some level of preparedness	
9. **Gathering mammoths’ fossils**	
*Associated resilience principles:* education and training; diversity; flexibility; community resources; resource development; collective action	
As a result of warmer temperatures in Northern Yakutia, permafrost thaw lead to accelerated coastal erosion and mammoth fossils (including carcasses, bones, tusks) are now more easily findable. This activity has been practiced by Indigenous peoples in the region since ancient times. Nowadays, an increasing amount of young people are getting involved in this activity in spite of the opinion of some of the most traditionalist sectors of northern communities who perceive this as an “threat to their worldviews.” Doing this could lead to opening the “underworld” and therefore, this could bring misfortune to the family of the gatherer	INTERVIEW 21 QUOTE 9–26: “It gets warmer and the tusks come out, they stick out. If it wouldn’t get warmer, the tusks would have been buried there I guess. So it gets warmer, and the tusks appear on the surface, so they take them out.” INTERVIEW 27 QUOTE 31–1: “Since permafrost thaws, bones and tusks come out in this way. And people are drawn for easy ruble, easy money and go (…) Well, I think that this does not affect me personally in any way. But it affects the lifestyle of the northern people. Previously, mammoth bones were never mind. For the older generation, this is a very big sin—if you take something from the earth. You can get food, you can get furs, you can get berries. Because man and nature are interconnected: you give to nature and nature gives you. But the remains must lie in the ground, buried. The remains cannot be touched according to our beliefs.”
Nowadays, some Indigenous peoples sell these types of remains to get an extra source of income for them. In addition, indigenous people (including small-numbered indigenous peoples) can gather these remains while conducting their traditional economic activities in specific designated areas. Laws stipulate that they can do it to gain extra income, for the protection of their indigenous rights or to limit uncontrolled and illegal gathering in the hard-to-reach lands of the Republic of Sakha. Despite the negative opinion of some sectors of local communities and the challenges for gatherers due to grays areas in the laws (such as the “one license, one sample” rule^a^), this activity can reinforce the overall resilience of future generations in the North. Nowadays, gatherers try to reconcile with their traditions, and after gathering samples from the soil, undertake a ritual in order to apologize for disturbing a dead soul. In general, they leave jewelry made of valuable beads in exchange to what they took from nature. Practicing this activity allow them to make ends meet and to adapt to the increasingly unstable socio-economic and environmental reality they are submerged in	
10. **Mobility**	
*Associated resilience principles:* self-determination; adaptation; action; decision-making; buffering	
In some cases, mobility emerged in locals’ narratives to explain how in the past—particularly after the Soviet breakup—they moved to remote places such as Moscow or Ukraine looking for better living opportunities, but eventually came back to the North (see interview 22 quote 21–2). More job opportunities and entertainment options elsewhere were not enough to maintain these respondents away from their settlements where they have strong social bonds and a sense of familiarity. In this case, the freedom of move implicitly allowed certain residents to revalue aspects related to their sense of belonging: family, friends and the unique climatic and environmental conditions are meaningful elements and they cannot live without them. For people living from traditional activities, who spend most of their time outdoors and live in synchronicity with natural cycles, trying to adapt to big urban areas can be burdensome	INTERVIEW 22 QUOTE 21–2: “No, we don't plan (to leave). I left once to Moscow and I didn't like it. 10 years ago I was offered to move and work in Moscow. I stayed there for two years and I didn't like it. I cannot adapt, sleep calmly. This [constant] movement [around]… I cannot adapt (…) [concerning the future of my children after they finish their studies] It will be their choice. I'll just teach them everything that I know. It is like this (sighs). Everybody disappears. I am concerned. But it doesn’t depend only on us” INTERVIEW 7 QUOTE 9: “I have been living in Tiksi since the year 83 all the time. I came from Baku, Azerbaijan. I arrived with three children, two more were born here. Two children are here, three in St. Petersburg. It looked like I would come here only for seasonal work in the 80s. In general, here I worked on rafting. Seasonally, there used to be a lot of people here. And the port worked, and rafting worked (…) I worked in “Tiksi Building” here. They built houses, they built a settlement
Mobility at a national and regional scale also allowed the arrival of “newcomers” in order to develop the Arctic region. For some, this was a temporary stay since they would eventually go back to their places of origin. During the Soviet Union, young professionals could benefit from Komsomol travel tickets that aimed at providing them with working opportunities in remote areas from the Russian territories (see interview 7 quote 9). Civil servants and inhabitants living in the Arctic also had access to early retirement, which was an economic way to encourage people to live there	
Daily mobility to other settlements, districts or regions is equally vital to work, practice traditional activities, study, or for leisure. This provides people the opportunity to obtain certain benefits (like traditional food or increased well-being) in other places. The freedom of movement allows them to choose what they deem best for their lives. This can also be interpreted as an adaptive strategy to leave places that no longer reunite the elements that they need in order to live in alignment with their worldviews, needs and priorities	

^a^“One sample” refers to one fossil found. Since mammoths lived in large groups, nowadays most fossil remains appear in large amounts and not individually. This is why this legal rule is considered by gatherers to be in misalignment with real-life conditions

## Discussion

These results provide a concrete example of how Resilient Community Development as a resilience framing (Wardekker (2021) can also be interpreted and formulated from a Russian Arctic perspective and thus beyond urban settings. These results also contribute to the scientific understanding of community resilience and reduce the overall conceptual vagueness by focusing on the specificities of these two coastal settlements. Despite the challenges that residents from Tiksi and Bykovsky are currently encountering, they are far from being powerless or deprived of agency. Although respondents do not seem to conceive their life experiences through the lenses of “resilience,” their ancestors lived in these territories for centuries and transferred to them multiple skills and capacities to thrive in such harsh climatic and environmental conditions. This can inform us on how to be resilient from an Arctic perspective in a rapidly changing twenty-first century. In fact, using local narratives as an entry point allowed me to bring to the surface in an explicit way multiple elements that are associated with how the local residents cope and desire to keep coping in the future with accelerated climatic, environmental and socio-economic changes. Despite the benefits of using this framework, it is possible to argue that it is not perfect and also presents limitations: for instance, it provides limited access to the local cosmology, leading to possible gaps in the ontological understanding of issues of local relevance.

**Discussion 1:** **the Resilient Community Development analysis calls for a focus on both the non-material and material dimensions of changes at the community level.**

These results reveal that the concept of resilience is strongly conditioned by normative issues and therefore, it is necessary to delve into every element that is associated to the specific social configuration of the settlements of Tiksi and Bykovsky. The way people perceive changes, the aspects that they deem meaningful and desirable for their lives, their settlements and future generations, as well as the desired actions to be taken are—and should be—elicited from within. The ten codes that emerged from the qualitative analysis (“solidarity,” “traditional activities,” “being local,” “young population,” “adaptation,” “share and support network,” “nostalgia and Soviet times,” “gathering of mammoths’ fossils,” “mobility” and “place attachment”) suggest that having a “common memory” associated to historical processes, specific ways of interacting with the surrounding environment, territories and other people, are key elements that not only shape the social configuration but also reinforce identity processes. In this sense, codes facilitated capturing of non-material elements that are important for northern residents to withstand rapid changes in a context of remoteness, harsh climatic conditions and limited financial support. By regrouping these codes, it was possible to identify broader patterns that inform us about: (1) how they actively adapt to changes on a daily basis, (2) who plays a key role at a local and regional scale regarding traditional food—which is at the core of the culture and identity in the Republic of Sakha—and knowledge transfer, and (3) what are the shared feelings, values and experiences that help locals counterbalance the negative effects of the reality they live in. These specific elements should not only enrich the western scientific understanding of community resilience, but also be translated into policies that are rooted in a deeper understanding of social, cultural and identity issues as experienced and communicated by local residents. It is here suggested that, if climate change and its impacts can lead to a forced restructuration of cultural features (such as the traditional activities) and a socio-cultural fragmentation (for example, due to the differences of perspective between generations regarding gathering mammoth fossils and living traditionally), identifying and analyzing the aforementioned non-material aspects could also have the opposite effect: to unveil and reinforce local forms of agency, adaptive capacities and resilience. Being aware of these capacities can enhance both individual and community strengths, facilitate coordinated action from within and for shared goals. This is the first contribution of MY (instead of OUR) results to the broader debates on resilience.

**Discussion 2:** **the Resilient Community Development analysis calls for a focus on social ties, reciprocity networks, on many scales**.

The forms of resilience that emerged from the narratives of residents from Tiksi and Bykovsky are deeply rooted in strong social ties within these communities with long-developed traditions of sharing and supporting each other. Local respondents pointed out that supporting each other is essential to survive in these latitudes. I argue that developing the existing relationships between the codes that emerged from narratives and the different resilience principles can facilitate the operationalization, application and reinforcement of resilience while respecting the concerns, desires and priorities of local residents.

Extending this analysis to a district or regional level can enhance the understanding of social issues and structures that exceed the formal administrative boundaries of these settlements but that are equally relevant to explore how community resilience might evolve. For example, in spite of the geographical remoteness of Tiksi and Bykovsky, non-material forms of support such as the share and support network (Doloisio 2022) play a key role at a local and regional scale. For Northerners, being part of these networks increases their sense of security and belongingness since they know that they can—when needed- rely on their relatives and friends. These implicit dynamics exceed formal administrative boundaries and shape the relationship between Northern residents (“villagers”) and urban dwellers at a regional scale. Although this exceeds the scope of this research, it might also be important to cross-examine the possible long-term effects of globalization within these coastal Russian Arctic settlements. For example, increased access to social media and technologies could facilitate the exercise of traditional activities, but this could also trigger the opposite effect by fostering the increasing disinterest from younger generations in living traditionally. Similarly, if climate change facilitates access to the North, how could globalization affect the bonds between northern residents and traditional food, which is also a key element of culture and identity in the Republic of Sakha. The relevance of the aforementioned multiscale linkages is a second fundamental contribution to the broader debate on resilience.

**Discussion 3:** **the Resilient Community Development analysis calls for a consideration of the geopolitical interest and socio-economic development of the Russian Arctic Zone.**

Narratives show that a strong sense of belonging, place attachment and nostalgia are shared by many inhabitants from Tiksi and Bykovsky. These feelings explain people’s reluctance to leave their settlements in spite of the challenges that they face on a daily basis. Living in this area means having the possibility to continue existing in the world in very specific ways: they are “locals,” they are highly mobile, they are active members of the share and support networks, they practice traditional activities and share strong feelings of nostalgia toward the Soviet days, among others. Analyzing the geopolitical situation in the Russian Arctic exceeds the scope of this research. However, the aforementioned elements demonstrate that local residents’ feelings—which are associated to how they perceive liveability and what the future possibilities for Northern settlements are, are closely linked to local forms of community resilience. Such elements must therefore be linked with the political situation of the Russian Arctic Zone. Due to its geographical location, Tiksi has been seen as a strategic town for the socio-economic development of the Russian Arctic. Considering the ongoing war with Ukraine and its international consequences, it is possible to infer that this will only reinforce the pre-existing interest of Russia in controlling and developing this region through federal and regional policies. Before the February 24th, 2022, a few guiding documents demonstrated such interest: the “Strategy for socio-economic development of the Arctic Zone of the Republic of Sakha until 2035” was signed in 2019 and aimed at promoting investments to modernize the northern transport system, to improve the internet connection and create more commercial opportunities based on social responsibility (while preserving the traditional lifestyle, culture and traditions of the Northern indigenous peoples), to improve the education and health systems and to promote Arctic tourism. More recently, in 2020, the Ministry of Arctic Development and the affairs of the People of the North of the Republic of Sakha, in collaboration with the Administration of the Bulunsky District and the citizens, they developed the “Comprehensive Plan of Development of Tiksi for the Period up to 2025” (Kondrateva 2017). In October 2020, Vladimir Putin signed the Strategy for the Development of the Russian Arctic Zone and National Security up to 2035. Although these documents do not specifically address climate change, they demonstrate the interest in accelerating the process of socio-economic development in the region. This could not only improve the quality of life, but most importantly, it would provide northern residents the freedom to live in alignment with their desires. The continued development of the region could lead to the enhancement of local forms of resilience, especially if we consider that many respondents openly manifested their desire and hope to “live like in the Soviet days” again. This historical period is associated by many respondents to regional prosperity and a stable socio-economic reality.

## Conclusion

This article demonstrates that using the concept of community development resilience as a frame is helpful to conceptualize life experiences from communities living in remote areas and explore how they cope with multifactorial changes and upheavals. I have used a qualitative narrative-centered analysis to reveal that material and non-material dimensions of changes should be given equal attention when analyzing community-level resilience, this with a focus on local norms. My results also reveal that remote communities’ social ties and reciprocity networks are central to the local understanding of what it means to cope with changes.

These results refer to a specific context, the Russian Arctic. Yet they also inform the broader resilience discourse. First, narrative-centered methodologies give access to the variety of local manifestations of community-level resilience. Secondly, while many environmental changes seem ab initio to relate to the materiality of the world, local communities are as dependent on the non-material dimensions for the development of the impacts that they perceive. Thirdly, this paper illustrates, and I believe necessarily, that quantitative equations or models per se cannot capture the totality of human experiences when it comes to environmental changes. In fact, these case studies showed that each of the elements, processes and stakeholders involved is intertwined within a complex, reactive and constantly fluctuating network of relationships. By focusing on experiential accounts that were expressed as everyday life narratives regarding feelings, shared memories, experienced challenges and life choices, it was possible to obtain a deeper understanding of the ongoing social, cultural and environmental upheavals that exist at local, district and regional scales.

## Supplementary Information

Below is the link to the electronic supplementary material.

Supplementary file1 (PDF 967 kb)

## Footnotes

1 In the Russian legislation, the concept of “indigenous peoples” does not exist. Instead, there are ethnicities. One of them is the “indigenous small-numbered peoples of the North, Siberia and the Far East”(Кopeнныe мaлoчиcлeнныe нapoды Ceвepa, Cибиpи и Дaльнeгo Bocт oкa Poccийcкoй Фeдepaции” in Russian). This category is applicable to those individuals who: (1) live on the territory of traditional occupancy of their ancestors—while maintaining traditional lifestyles, (2) numbering less than 50.000 persons, and (3) who consider themselves as ethnic communities with a distinct self-identity (Sikora 2020). The Sakha, as many other indigenous groups, are legally excluded from this category as they exceed the 50.000 individuals.
