# Supplementary material for: Exploring community resilience through Arctic residents’ narratives in the Republic of Sakha (Russia)
Source: Ambio. 2024 Oct 10;54(1):135–50. doi: 10.1007/s13280-024-02071-y (PMC11607273; doi:10.1007/s13280-024-02071-y)
Supplement: Supplementary file 1 — Supplementary file1 (PDF 967 kb) [file 13280_2024_2071_MOESM1_ESM.pdf]

Ambio

Supplementary Information
This supplementary information has not been peer reviewed.

Title: **Interview framework for residents from Tiksi and Bykovsky (Bulunsky District, Republic of Sakha).**

NUNATARYK Project: Permafrost thaw and the changing Arctic coast, science for socio-economic adaptation
Проект Нунатарюк: таяние вечной мерзлоты и изменение арктического побережья, наука в целях социально-экономической адаптации

Introduction to the interview People from Bulunsky District, (Republic of Sakha)	Вводная часть к опросу населения Булунского района (Республики Саха)
**First we begin by clarifying the project and our goals.**	**Первым делом мы объясним суть проекта и наши цели.**
In continuity with the interviews that were conducted in Yakutsk in June and July 2018, you are invited to participate in an interview about permafrost, its importance, in all dimensions of life in Sakha republic. We invite you to narrate your personal experience and your opinions. You help this research a lot with your knowledge.	В рамках научного проекта Nunataryuk в июле 2018 г. в Якутске проводились опросы, касающиеся темы таяния вечной мерзлоты, ее важности во всех аспектах жизни в Республике Саха (Якутия). В продолжение этих опросов, предлагаем Вам рассказать о своем личном опыте и поделиться своими взглядами. Благодаря своим знаниям Вы окажете неоценимую помощь этому исследованию.
**About the Nunataryuk project**	**О проекте Nunataryuk**
Most human activity in the Arctic takes place along permafrost coasts and these coasts have become one of the most dynamic ecosystems on Earth. Permafrost thaw is exposing these coasts to rapid change, change that threatens the rich biodiversity, puts pressure on communities and contributes to the vulnerability of the global climate system. NUNATARYUK project will determine the impacts of thawing coastal and subsea permafrost on the global climate, and will develop targeted and co-designed adaptation and mitigation strategies for the Arctic coastal population in several regions (Northwest Canada, Svalbard, Greenland and Russia).	Основная часть человеческой деятельности в Арктике происходит вдоль берегов вечной мерзлоты, и эти берега стали одной из наиболее динамичных экосистем на Земле. Таяние вечной мерзлоты подвергает эти берега стремительным изменениям, угрожающим богатому разнообразию, оказывающим давление на общины и способствующим повышению уязвимости глобальной климатической системы. Проект NUNATARYUK позволит определить последствия таяния прибрежной и подводной вечной мерзлоты для глобального климата и разработать целевые и согласованные стратегии адаптации и минимизации последствий для прибрежного населения Арктики в нескольких регионах (Северо-Западная Канада, Шпицберген, Гренландия и Россия).
**About Our Team**	**О нашей исследовательской группе**
The main objective of the project is to study the socio-economic impacts of permafrost thawing for the coastal Arctic regions of Yakutia (in particular, the Bulunsky District). The researchers will conduct face-to-face interviews with local inhabitants in order to understand their perception on how permafrost thaw affects their	Основная задача в рамках проекта состоит в исследовании социально-экономических последствий таяния вечной мерзлоты для прибрежных арктических регионов Якутии (в частности, Булунского района). В ходе исследования будут проведены интервью с местными жителями с целью понимания их

everyday lives and activities.<br><br>Through individual community-oriented interviews and meetings with stakeholders, local narratives about permafrost thawing will be collected. They will be analyzed in the context of coastal and climate risk management, as well as economics and natural resource management, the development of coastal communities.<br><br>This project is also an opportunity to sustain the well-established relationship between Universite de Versailles-Saint-Quentin-en-Yvelines and the Northeastern Federal University.	восприятия того, как таяние вечной мерзлоты влияет на их повседневную жизнь и деятельность.<br><br>Через индивидуальные интервью, ориентированные на сообщества, и встречи с заинтересованными сторонами, будут собраны местные рассказы о таянии вечной мерзлоты. Они будут проанализированы в контексте управления прибрежными и климатическими рисками, а также экономики и управления природными ресурсами, развития прибрежных общин.<br><br>Данный проект также представляет собой возможность сохранения прочно установившихся отношений между Университетом Версаль Сен-Кентен-ан-Ивелин и Северо-Восточным федеральным университетом.
**Potential Benefits and Risks:**	**Потенциальные выгоды и риски:**
Your participation in this research study will include a commitment of your time; however, if you decide to participate, your willingness to share your knowledge could provide valuable insights towards understanding current challenges and opportunities for community development in regards to thawing permafrost. This information can eventually inform decisions made by policymakers collaborating across the Circumpolar north.	Участие в данном исследовании потребует от вас затрат личного времени; с другой стороны, приняв решение об участии и выразив готовность поделиться своими знаниями, вы сможете внести ценный вклад в понимание текущих проблем и возможностей для развития сообщества в отношении таяния вечной мерзлоты. Эта информация в конечном итоге позволит создать основу для решений, принимаемых политиками относительно сотрудничества в арктических регионах.
**Confidentiality:**	**Конфиденциальность:**
All raw interview data will remain confidential. Only the researchers who conduct this interview, as well as the researchers who are working on the same topic in other study regions of NUNATARYUK, will have access to the raw data (names and contacts listed at the end of this form). I am asking your permission to audio-record this interview to aid in note taking. If you permit recording, the recording will not be shared with anyone outside NUNATARYUK. Names will not be attached to information and views you have shared. All interview notes, audio files as well as transcripts will be kept in a secure file cabinet, to which only the mentioned researchers have access, as well as on a password-protected computer.<br><br>Your contribution will be anonymous and remains	Все исходные данные интервью останутся конфиденциальными. Доступ к необработанным данным (именам и контактам, перечисленным в конце этой формы) будут иметь только исследователи, проводящие данное интервью, а также исследователи, работающие над этим проектом (NUNATARYUK). В связи с этим просим Вашего разрешения на аудиозапись этого интервью в целях облегчения регистрации данных. В случае вашего разрешения на ведение аудиозаписи эта запись не будет передана ни одному лицу за пределами проекта NUNATARYUK. Представленная вами информация и взгляды будут анонимными. Все данные интервью, аудиофайлы, а также их расшифровка будут храниться в защищенном файловом шкафу, доступ к которому имеют лишь

confidential. In publications either no name or pseudonyms will be used. All members of the research team are obliged to keep confidentiality and are not allowed to release the identity of an interview partner. Only the internal research team has access to your interview. We also are asking that you keep the discussion confidential; in case of a group interview please do not share with anyone outside of the interview what was said by other participants. You may tell people about the general topics of discussion, and share your own views, but not any information that reveals what was said by anyone else.	вышеупомянутые исследователи, а также на защищенном паролем компьютере.<br><br>Ваш вклад будет анонимным и останется конфиденциальным. В публикациях не будут использованы ни имена, ни псевдонимы. Все члены исследовательской группы обязаны соблюдать конфиденциальность и не имеют права разглашать личные данные участника интервью. Мы также просим вас сохранять конфиденциальность обсуждения.
Voluntary Participation:	Добровольное участие:
Participation in this study is entirely voluntary. You may refuse to participate in this now or at any time during or after the interview or focus group. You may leave the interview and/or group discussion at any time, or simply not reply to any question you do not wish to answer. The interview will take between half an hour and one hour and shall not exceed one and a half hours.	Участие в этом исследовании носит абсолютно добровольный характер. Вы можете отказаться от участия в нем сейчас или в любой момент во время или после интервью или заседания тематической группы. Вы можете покинуть интервью и (или) групповое обсуждение в любой момент, либо можете просто не отвечать на любой вопрос, на который не желаете отвечать. Интервью займет от получаса до часа и не должно длиться более полутора часов.
Thank you very much for your participation!	Большое вам спасибо за участие!
**Interview framework – scoping exercise**	**Рамки интервью – обзор таяния вечной мерзлоты**
Please briefly introduce yourself for the record. (Name, age, where were you born, how long have you lived in Tiski/Bykovskiy, what are you currently doing?	Пожалуйста, вкратце расскажите о себе (Имя, возраст, место рождения, как долго вы живете в Тикси/Быковском, чем занимаетесь в настоящее время?)
My first question and most of the question will be quite general. There are no wrong answers. We are interested in YOUR experience, in YOUR opinion.	Пояснение. Все вопрос носят общий характер. Никакие ответы не могут считаться неправильными. Нас интересует именно ВАШ опыт и ВАШЕ мнение.
Question 1: What can you tell me about life in your town/village? Feel free to tell me about anything you consider important for you.	Вопрос 1: Что вы можете рассказать о жизни в вашем поселке? Пожалуйста, расскажите, что считаете важным для себя.
Prompt to be used only if the interviewee does not mention this at all:	Наводящие вопросы используются только в том случае, если интервьюируемый совершенно не упоминает следующие аспекты:
Cultural life ☐	Культурная жизнь
Education ☐	Образование
Health ☐	Здоровье
Traditions ☐	Традиции
Well-being in general ☐	Благосостояние в целом
Natural resources ☐	Природные ресурсы
Question 2: According to you and your own experience, what are the most important changes	Вопрос 2: По вашему ощущению и опыту, каковы наиболее важные изменения,

that your community is facing today?	происходят сегодня в вашем поселке?
Prompts	Наводящие вопросы
Challenge to live in your city/village ☐	Проблемы жизни в вашем поселке
Daily activities ☐	Повседневная деятельность
Causes of the changes ☐	Причины изменений
Opinion about changes ☐	Мнение об изменениях
Most impacted community members ☐	Наиболее уязвимые слои населения
Question 3: According to you and your own experience, how is the climate evolving? Could you identify why?	Вопрос 3: По вашему ощущению и опыту, какие изменения происходят с климатом? Каким образом вы их определяете (замечаете)?
Prompts	Наводящие вопросы
Observed examples (temperatures, rain/snow falls, ice or snow texture, etc.) ☐	Примеры наблюдений (температура, количество дождя/снега, текстура льда или снега и т.д.)
Opinion on climate evolution ☐	Мнение об изменении климата
Unsafe situations related to climate ☐	Опасности, связанные с климатом
Question 4: From your experience, your everyday life, what is permafrost thaw? Can you describe it?	Вопрос 4: Исходя из Вашего опыты, по ощущениям из повседневной жизни, что такое таяние вечной мерзлоты? Можете ли вы его охарактеризовать?
Prompts	Наводящие вопросы
Observed ground changes …	Наблюдаемые изменения почвы…, …
Other issues …	другое
Question 5: According to you and your own experience, are the permafrost/frozen ground conditions changing? And if yes to what extent? How do you notice it? Does this impact you? If yes, how?	Вопрос 5: По вашим ощущениям и жизненному опыту, изменяется ли состояние вечной мерзлоты/мерзлого грунта? Если да, то в какой степени? Каким образом вы это замечаете? Влияет ли это на вас лично? Если да, то как?
Prompts	Наводящие вопросы
Coastal erosion ☐	Береговая эрозия (размывание/разрушение берегов)
Infrastructure collapse (building, roads, ports airports, bridges) ☐	Обрушение объектов инфраструктуры (зданий, дорог, морских портов, аэропортов, мостов)
Cultural loss ☐	Утрата культуры
Food production ☐	Производство пищи
Local authority actions to assess impacts of permafrost thaw ☐	Действия местных властей по оценке последствий таяния вечной мерзлоты
Question 6: Have you or your relatives already “suffered” or experienced an “unsafe” situation that can be linked to permafrost thaw/melting ground? If so, would you mind describing it?	Вопрос 6: Лично вы или кто-либо из ваших родственников испытывали влияние таяния вечной мерзлоты (негативное или положительное)? Если да, не могли бы описать такую ситуацию?
Prompts	Наводящие вопросы
Risky situations ☐	Рискованные ситуации
Solutions to these risks (your own skills, agency) ☐	Преодоление этих рисков (ваши собственные умения, возможности влияния)
Question 7: What do you expect from the future? What are your main concerns and challenges for the future? What can you tell us about the quality of life in the Arctic region?	Вопрос 7: Что вы ожидаете от будущего? Какие основные беспокойства и трудности вы связываете с будущим? Как Вы оцениваете качество жизни в

	**арктическом районе?**
Prompts	Наводящие вопросы
Life in Tiksi in the future ☐	Жизнь в Тикси сейчас и в будущем
	Насколько Вы удовлетворены качеством предоставляемых услуг в образовании, в здравоохранении, в связи, в проведении досуга и др.?
Tools/skills/capacities to face future risks or to adapt:<br><br>- less/best prepared activity (housing, health, mobility, traditional profession, ...) ☐<br><br>- tools (quotas, taxation or other incentives) ☐	Средства/навыки/возможности преодоления будущих рисков и/или адаптации:<br><br>- менее/наиболее подготовленная деятельность (жилье, здравоохранение, мобильность, традиционная профессия, ...)<br><br>- инструменты (квоты, налогообложение или другие стимулирующие факторы)
Adaptation (at individual, and Tiksi, Bulunsky district, Yakutia, and national scales) ☐	Адаптация (на индивидуальном уровне, в масштабах Тикси, Булунского района, Якутии и в российском масштабе)
Cooperation between these 5 scales ☐	Взаимодействие между этими пятью масштабами
**Question 8: Are you planning to move? What reasons may influence your decision? What do you expect for your children once they have finished their studies?**	**Вопрос 8: Планируете ли Вы переехать? Какие причины могут повлиять на Ваше решение?**<br><br>**Как Вы видите будущее своих детей после получения образования?**
Prompts	Наводящие вопросы
**Question 9: How many people in your household? Precise evaluation of income and expenditure flows for one month? Precise evaluation of sources of food and other primary necessities for one month? Connection with permafrost?**	**Вопрос 9: Сколько человек у вас в семье (домашнем хозяйстве)? Каковы Ваши ежемесячные доходы и расходы? Можете назвать объем источников продовольствия и других предметов первой необходимости на один месяц? Если их связь с оттаиванием вечной мерзлотой?**
Prompts	Наводящие вопросы
Living/professional activity ☐	Жизнедеятельность/профессиональная деятельность ☐
Estimated savings per month ☐	Примерная сумма сбережений (накоплений) за один месяц ☐
Number of children under 18 years old living with you (or upper 18 but unemployed) ☐	Число живущих с вами детей младше 18 лет (либо неработающих детей старше 18 лет) ☐
**Question 10: Is there anything else I did not ask and you think I should know to improve my understanding of permafrost thaw? Is there anything you would like to ask us?**	**Вопрос 10: Есть ли вопросы к нам? Может быть, мы пропустили что-то важное? Что бы Вы хотели от нас услышать?**
